# A Retrospective Cohort Analysis of Mental Health-Related Emergency Department Visits Among Veterans and Non-Veterans Residing in Ontario, Canada: Une analyse de cohorte rétrospective des visites au service d’urgence liées à la santé mentale parmi les vétérans et non-vétérans résidant en Ontario, Canada

**DOI:** 10.1177/07067437231223328

**Published:** 2024-01-05

**Authors:** Kate St. Cyr, Peter Smith, Paul Kurdyak, Heidi Cramm, Alice B. Aiken, Alyson Mahar

**Affiliations:** 1 Dalla Lana School of Public Health, 7938 University of Toronto, Toronto, ON, Canada; 2 30678 Institute for Work and Health, Toronto, ON, Canada; 3 ICES Central, Toronto, ON, Canada; 4 Temerty Faculty of Medicine, 7938 University of Toronto, Toronto, ON, Canada; 5 Institute for Health Policy, Management, and Evaluation, 7938 University of Toronto, Toronto, ON, Canada; 6 Centre for Addiction and Mental Health, Toronto, ON, Canada; 7 School of Rehabilitation Therapy, 4257 Queen's University, Kingston, ON, Canada; 8 3688 Dalhousie University, Halifax, NS, Canada; 9 School of Nursing, Queen's University, Kingston, ON, Canada

**Keywords:** emergency departments, health services, mental health, veterans, services d'urgence, services de santé, santé mentale, vétérans

## Abstract

**Objectives:**

Emergency departments (EDs) are a vital part of healthcare systems, at times acting as a gateway to community-based mental health (MH) services. This may be particularly true for veterans of the Royal Canadian Mounted Police who were released prior to 2013 and the Canadian Armed Forces, as these individuals transition from federal to provincial healthcare coverage on release and may use EDs because of delays in obtaining a primary care provider. We aimed to estimate the hazard ratio (HR) of MH-related ED visits between veterans and non-veterans residing in Ontario, Canada: (1) overall; and by (2) sex; and (3) length of service.

**Methods:**

This retrospective cohort study used administrative healthcare data from 18,837 veterans and 75,348 age-, sex-, geography-, and income-matched non-veterans residing in Ontario, Canada between April 1, 2002, and March 31, 2020. Anderson–Gill regression models were used to estimate the HR of recurrent MH-related ED visits during the period of follow-up. Sex and length of service were used as stratification variables in the models.

**Results:**

Veterans had a higher adjusted HR (aHR) of MH-related ED visits than non-veterans (aHR, 1.97, 95% CI, 1.70 to 2.29). A stronger effect was observed among females (aHR, 3.29; 95% CI, 1.96 to 5.53) than males (aHR, 1.78; 95% CI, 1.57 to 2.01). Veterans who served for 5–9 years had a higher rate of use than non-veterans (aHR, 3.76; 95% CI, 2.34 to 6.02) while veterans who served for 30+ years had a lower rate compared to non-veterans (aHR, 0.60; 95% CI, 0.42 à 0.84).

**Conclusions:**

Rates of MH-related ED visits are higher among veterans overall compared to members of the Ontario general population, but usage is influenced by sex and length of service. These findings indicate that certain subpopulations of veterans, including females and those with fewer years of service, may have greater acute mental healthcare needs and/or reduced access to primary mental healthcare.

## Résumé

**Objectifs:**

Les services d’urgence (SU) sont une partie essentielle des systèmes de santé, qui agissent parfois à titre de passerelle aux services de santé mentale (SM) communautaires. Ceci s’avère particulièrement vrai pour les vétérans de la Gendarmerie royale du Canada (GRC) qui ont été libérés avant 2013 et des Forces armées canadiennes (FAC), car ces personnes font la transition de la couverture des soins de santé fédérale à la couverture provinciale lors de leur libération et peuvent utiliser les SU en raison des délais pour obtenir un fournisseur de soins de première ligne. Nous cherchions à estimer le rapport de risque (RR) des visites au SU liées à la SM entre vétérans et non-vétérans résidant en Ontario, Canada : (1) dans l’ensemble; et selon (2) le sexe; et (3) la durée du service.

**Méthodes:**

Cette étude de cohorte rétrospective a utilisé des données de santé administratives de 18,837 vétérans et 75,348 non-vétérans appariés selon l’âge, le sexe, la géographie, et le revenu résidant en Ontario, Canada entre le 1^er^ avril 2002 et le 31 mars 2020. Des modèles de régression Anderson–Gill ont servi à estimer le RR récurrent des visites au SU liées à la SM durant la période du suivi. Le sexe et la durée du service ont servi de variables de stratification dans les modèles.

**Résultats:**

Les vétérans avaient un RR ajusté (RRa) plus élevé de visites au SU liées à la SM que les non-vétérans (RRa,1,97, IC à 95%,1,70 à 2,29). Un effet plus fort était observé parmi les femmes (RRa, 3,29; IC à 95%, 1,96 à 5,53) que chez les hommes (RRa, 1,78; IC à 95%, 1,57 à 2,01). Les vétérans qui avaient servi de 5 à 9 ans avaient un taux d’utilisation plus élevé que les non-vétérans (RRa, 3,76; IC à 95%, 2,34 à 6,02) tandis que les vétérans qui ont servi pendant plus de 30 ans avaient un taux plus faible comparé aux non-vétérans (RRa, 0,60; IC à 95%, 0,42 à 0,84).

**Conclusions:**

Les taux des visites au SU liées à la SM sont plus élevés chez les vétérans dans l’ensemble comparativement aux membres de la population générale de l’Ontario, mais l’usage est influencé par le sexe et la durée du service. Ces résultats indiquent que certaines sous-populations de vétérans, y compris les femmes et ceux ayant moins d’années de service, peuvent avoir des besoins de soins de santé mentale aigus plus graves et/ou un accès réduit aux soins de santé mentale de première ligne.

## Introduction

Exposure to potentially psychologically traumatic events while employed in military careers can lead to the development or exacerbation of mental health (MH) concerns.^1–4^ Military veterans in the United States (US), Canada, the United Kingdom (UK), and Australia are more likely to experience depressive and anxiety disorders than members of the general population^5–11^; however, the risk of adverse MH outcomes may vary by nation, sex, and length of service. For example, female veterans may differentially experience psychologically traumatic military-related events, such as military sexual trauma, compared to their male counterparts,^12^ potentially leading to higher rates of MH conditions^13–15^ and mental healthcare (MHC) use^15,16^; while early service-leavers have been shown to have a greater risk of MH disorders than individuals who serve for longer periods.^17^

MHC use is also influenced by the availability and affordability of appropriate services,^18,19^ awareness and willingness to seek MHC,^20–23^ and ease of access to MHC.^23,24^ Emergency departments (EDs) combine accessibility and the capability to address MH crises, meaning they may act as the first point of contact for community-based MHC.^25–27^ Following release, veterans in the UK, Australia, and Canada transition from specialized military-specific healthcare to a publicly funded civilian health insurance plan,^7,28–30^ and may experience delays obtaining a civilian primary care provider (PCP) or find themselves rostered with a PCP who is unfamiliar with military culture/healthcare needs^23^ and therefore rely on EDs for MHC, at least for a period of time. In the US, where some veterans have access to Veterans Affairs-provided healthcare while others rely on private insurance or state-sponsored plans,^31^ accessibility and affordability act as barriers to community-based healthcare, leading to increased reliance on EDs for MHC.^19^

Together, existing research suggests that veterans may have an increased need for accessing MHC through EDs compared to non-veterans, which may be further influenced by sex and length of service. A comprehensive investigation of the rates and timing of MH-related ED visits is necessary for understanding how veterans in Ontario interact with the provincial healthcare system for acute MH needs, and whether sex- and length of service-based gaps in access to community-based MHC may exist. The objectives of this research are to compare MH-related ED visit rates between veterans and non-veterans residing in Ontario between 2002 and 2020: (1) overall; and by (2) sex; and (3) length of service.

## Methods

### Study Design

We employed a matched, retrospective cohort design of veterans and non-veterans residing in Ontario, Canada between April 1, 2002, and March 31, 2020. We used administrative healthcare data held at ICES (previously known as the Institute for Clinical Evaluative Sciences), a not-for-profit research institute whose legal status under Ontario's health information privacy law allows it to collect and analyse healthcare and demographic data, without consent, for health system evaluation and improvement.

### Study Population

The study population consisted of Canadian Armed Forces (CAF) Regular Force and Royal Canadian Mounted Police (RCMP) veterans who took up residence in Ontario following the completion of their service, and a matched cohort was drawn from members of the Ontario general population. Veterans were identified in the data holdings via a Ministry of Health (MOH)-supplied list of anonymized individuals with an administrative veteran identifying code, which was then linked to ICES Key Numbers (IKNs), a unique encoded identifier.

### Inclusion/Exclusion Criteria

Veterans were included in the current study if they registered for the Ontario Health Insurance Plan (OHIP) between April 1, 2002, and March 31, 2019, for CAF veterans; and April 1, 2002, and December 31, 2013, for RCMP veterans, since in 2013 the RCMP transitioned all its members to provincial healthcare plans so they no longer transitioned from the federally funded healthcare system at release. Veterans who: (1) were 16 years of age or younger at the time of recruitment into the Forces; or (2) had OHIP coverage at any time during their period of service were excluded from this study.

Non-veterans were eligible for inclusion in this study if they were alive at the time of the study index date (i.e., OHIP registration date for their matched veteran). Non-veterans were hard-matched to veterans at a rate of 4:1 on age at index (using birth year), sex, region of residence (1 of 14 Local Health Integration Networks [LHINs]), and median neighbourhood income quintile. LHIN and income quintile were determined using census data linked to postal code data. Non-veterans who had a long-term care or rehabilitation facility stay or received disability or income support payments prior to their assigned index date were not eligible for inclusion in the study given the decreased likelihood of employment during the matched veterans’ period of service.

### Data Sources

The current study used Ontario's healthcare administrative databases, which were linked using IKNs and analysed at ICES. Demographic data, including age, sex, region of residence, and neighbourhood median income quintile, were retrieved from the Registered Persons Database, while MH-related ED visits were identified using the National Ambulatory Care Reporting System (NACRS) database.

### Study Variables

#### Exposure Variable

The primary exposure was a dichotomous variable for veteran status (e.g., veteran/non-veteran).

#### Outcome Variable

The outcome of interest was an MH-related ED visit occurring between April 1, 2002, and March 31, 2020, as captured in the NACRS data. An ED visit was considered MH-related if the primary diagnostic code associated with the visit fell under one of the following diagnostic groupings, which were allocated using an established ICES approach: anxiety, deliberate self-harm, mood disorders, schizophrenia/other psychotic disorders, substance use, and other MH. The International Classification of Disease, Tenth Revision (ICD-10)^32^ codes included in each of these diagnostic groupings can be found in Supplemental Table S1. For individuals with >1 same-day MH-related ED visit, only the first same-day visit was included in the analyses.

#### Effect Measure Modifiers

Sex and length of service were included in the analyses as effect measure modifiers. For veterans, length of service was calculated, in years, using the CAF or RCMP service start and end dates supplied by the MOH. We categorized length of service as <5 years, 5–9 years, 10–19 years, 20–29 years, and 30+ years. The first 9 years of service were split into 2 distinct intervals to differentiate “early service leavers.” Non-veterans were placed in the same length of service stratum as their matched veterans. Interaction terms between (1) sex and veteran status and (2) length of service and veteran status were included in the regression model. As the interaction terms were statistically significant (*P* = 0.0253 and <0.001, respectively), stratified analyses were used to explore the effect of veteran status by sex and length of service.

#### Covariates

We included all matching variables, held at the index date, as covariates in the multivariable analyses. Age was categorized as <30 years, 30–39 years, 40–49 years, and 50+ years, while sex was dichotomized as male/female. We retained income as neighbourhood median income quintiles, and region of residence as a 14-level categorical variable based on postal code-assigned LHINs. We also included rurality and a number of major and minor comorbidities as additional covariates in the multivariable analyses.

Rurality was ascertained at the index date using Rurality Index of Ontario (RIO) 2008 scores, which account for a community's size, density, and proximity to healthcare services and range from 0 to 100.^33^ We used Lucas et al.'s^34^ RIO score categorizations: 0–3 (large, urban centres with access to advanced referral centres), 4–14 (urban centres with access to basic referral centres), 15–39 (small urban centres), 40–74 (rural communities), and 75+ (remote, rural communities).

A number of major and minor comorbidities were assessed at 1 year following the index date using the Johns Hopkins Adjusted Clinical Groups (ACG)® system for categorizing illnesses using the International Classification of Disease, Ninth Revision (ICD-9) and ICD-10 codes. ICD codes are assigned to 1 of the 32 ACG® System Aggregated Diagnosis Groups (ADGs), based on the aetiology, duration, and severity of the condition, diagnostic certainty, and involvement of specialty care.^35,36^ To minimize the risk of artificial inflation with the outcome, we excluded the 1 major and 2 minor psychosocial ADGs and summed the remaining ADGs in the first year of follow-up to create 2 count variables: number of major comorbidities (range = 0–7) and number of minor comorbidities (range = 0–22).

### Analytic Approach

We used standardized mean differences and variance ratios^37^ to compare the distribution of baseline characteristics in the veteran and non-veteran cohorts. Means and standard deviations were used to describe continuous variables, and frequencies and proportions were used to describe categorical variables. The mean cumulative function (MCF), a nonparametric function, which estimates the population's mean cumulative number of events over time,^38,39^ was estimated by veteran status and stratified by sex and length of service. We plotted the MCFs to visually compare the intensity of ED visits over the period of follow-up between groups.

We used Andersen–Gill (AG) models^40^ or Cox counting process models to capture recurrent ED visits. Unlike Poisson regression or other methods commonly used for count data, AG models not only consider the cumulative number of events but also consider the timing of each recurrent event.^41^ We used robust sandwich variance estimates to account for correlations induced by the matching approach used in the creation of the cohorts, as well as the dependencies between each individual's ED visits. Unadjusted and adjusted AG regression models were used to compare ED visit rates by veteran status overall and stratified by sex and length of service. The adjusted model included all matching variables (age at index [years], sex, LHIN, and median neighbourhood income quintile), rurality, and a number of major and minor comorbidities. For all analyses, we used 2-sided hypothesis tests, and *P*-values < 0.05 were considered statistically significant. Analyses were conducted using SAS statistical software v.9.4.^42^

## Results

### Cohort Description

A total of 18,846 veterans and 75,375 non-veterans were identified for inclusion in the cohort. Nine veterans were unable to be matched to 4 non-veterans each because of unique combinations of the matching variables; as such, each of these incompletely matched sets was excluded from the analysis for a final cohort of 18,837 veterans and 75,348 non-veterans.

The mean age of the individuals included in this study was 42.3 years; 84.5% were male (*n* [veterans] = 15,914; *n* [non-veterans] = 63,656). The average duration of follow-up from the index date was 7.85 years for veterans and 8.20 years for non-veterans. The baseline characteristics of veterans and non-veterans are presented in Table 1. Veterans were more likely to live in smaller urban centres and rural communities than non-veterans, and less likely than non-veterans to live in densely populated urban centres. A larger proportion of veterans had one or more major comorbidities than non-veterans (21.5% [*n* = 14,787] vs. 18.5% [*n* = 61,409]), and veterans were more likely than non-veterans to exit the cohort by moving out of province (12.2% [*n* = 2307] vs. 7.8% [*n* = 5839]).

**Table 1.** Baseline Characteristics, Standard Mean Differences, and Variance Ratios of Veterans Residing in Ontario and an Age-, Sex-, Geography-, and Income-Matched Non-Veteran Cohort.

	Veterans (*N* = 18,837)	Non-veterans (*N* = 75,348)	Std. mean diff	Var. ratio
Variable	Mean (*SD*)			
Age (years)	42.34 (10.73)	42.34 (10.72)	0.00	1.00
Years of follow-up	7.85 (5.15)	8.20 (5.07)	0.07	0.97
	*N* (%)			
Sex				
Male	15,914 (84.5)	63,656 (84.5)	0.00	4.00
Female	2923 (15.5)	11,692 (15.5)	0.00	4.00
Income quintile				
1 (lowest)	2029 (10.8)	8116 (10.8)	0.00	4.00
2	3133 (16.6)	12,532 (16.6)	0.00	4.00
3	4116 (21.9)	16,464 (21.9)	0.00	4.00
4	4873 (25.9)	19,492 (25.9)	0.00	4.00
5 (highest)	4686 (24.9)	18,744 (24.9)	0.00	4.00
LHIN				
Erie St. Clair	329 (1.7)	1316 (1.7)	0.00	4.00
South West	799 (4.2)	3196 (4.2)	0.00	4.00
Waterloo-Wellington	314 (1.7)	1256 (1.7)	0.00	4.00
HNHB	575 (3.1)	2300 (3.1)	0.00	4.00
Central West	143 (0.8)	572 (0.8)	0.00	4.00
Mississauga-Halton	250 (1.3)	1000 (1.3)	0.00	4.00
Toronto Central	214 (1.1)	856 (1.1)	0.00	4.00
Central	385 (2.0)	1540 (2.0)	0.00	4.00
Central East	459 (2.4)	1836 (2.4)	0.00	4.00
South East	4054 (21.5)	16,216 (21.5)	0.00	4.00
Champlain	9181 (48.7)	36,724 (48.7)	0.00	4.00
North Simcoe-Muskoka	1424 (7.6)	5696 (7.6)	0.00	4.00
North East	627 (3.3)	2508 (3.3)	0.00	4.00
North West	83 (0.4)	332 (0.4)	0.00	4.00
Rurality				
Missing	63 (0.3)	432 (0.6)	0.04	2.34
1 (most urban)	8891 (47.2)	39,889 (52.9)	0.11	4.00
2	3277 (17.4)	9233 (12.3)	0.15	5.35
3	3520 (18.7)	16,825 (22.3)	0.09	3.50
4	3031 (16.1)	8611 (11.4)	0.14	5.34
5 (most rural)	55 (0.3)	358 (0.5)	0.03	2.46
No. major ADGs				
0	14,787 (78.5)	61,409 (81.5)	0.08	4.48
1	3378 (17.9)	11,584 (15.4)	0.07	4.52
2+	672 (3.6)	2355 (3.1)	0.02	4.54
No. minor ADGs				
0	5796 (30.8)	26,038 (34.6)	0.08	3.77
1–2	7826 (41.5)	30,329 (40.3)	0.03	4.04
3–5	4511 (23.9)	16,365 (21.7)	0.05	4.28
6+	704 (3.7)	2616 (3.5)	0.01	4.29
Length of service				
<5 years	2805 (14.9)	–	–	–
5–9 years	2318 (12.3)	–	–	–
10–19 years	2843 (15.1)	–	–	–
20–29 years	6385 (33.9)	–	–	–
≥30 years	4486 (23.8)	–	–	–
Reason for end of follow-up				
Death	217 (1.2)	993 (1.3)	0.02	3.50
Loss of OHIP eligibility	2307 (12.2)	5839 (7.8)	0.15	6.01
End of study period	16,313 (86.6)	68,516 (90.9)	0.14	5.63

*Note*. Std. mean diff. = standard mean differences; var. ratio = variance ratio; LHIN = Local Health Integration Network; HNHB = Hamilton Niagara Haldimand Brantford; OHIP = Ontario Health Insurance Plan.

### MH-Related ED Visits

Among veterans, 5.7% (*n* = 1065) had a least 1 MH-related ED visit during the period of follow-up, compared to 3.7% of non-veterans (*n* = 2790). Descriptive ED use data are presented by veteran status overall and by sex (Table 2) and length of service (Table 3).

**Table 2.** MH-Related ED Use During Period of Follow-up (April 1, 2002 to March 31, 2020), by Veteran Status and Sex.

	Veterans			Non-veterans		
	Overall (*N* = 18,837)	Males (*n* = 15,914)	Females (*n* = 2923)	Overall (*N* = 75,348)	Males (*n* = 63,656)	Females (*n* = 11,692)
No. (%) with at least 1 MH-related ED visit	1065 (5.65)	876 (5.50)	189 (6.47)	2790 (3.70)	2365 (3.72)	425 (3.63)
Mean no. visits (*SD*)^a^	2.29 (4.43)	2.20 (2.96)	2.70 (8.36)	1.80 (2.25)	1.83 (2.34)	1.65 (1.66)
Range	0-109	0-40	0-109	0-39	0-39	0-14
Total no. MH-related ED visits	2440	1929	511	5021	4322	699
Total no. person years of follow-up^b^	147,807	126,467	21,340	617,750	523,515	94,285
Crude rate of MH-related ED visits^c^	0.17	0.15	0.24	0.08	0.08	0.07
Crude rate ratio (veterans vs non-veterans; ref. group = non-veterans)	2.13	1.88	3.43	1.00	1.00	1.00

*Note*. MH = mental health; ED = emergency department; SD = standard deviation; Ref. group = reference group.

^a^ Among individuals with at least 1 MH-related ED visit.

^b^ Rounded up to the nearest whole number.

^c^ Per 10 person-years of follow-up.

**Table 3.** MH-Related ED Use During Period of Follow-up (April 1, 2002, to March 31, 2020), by Veteran Status and Length of Service.

	Veterans					Non-veterans				
	<5 years (*n* = 2805)	5–9 years (*n* = 2318)	10–19 years (*n* = 2843)	20–29 years (n = 6385)	30+ years (*n* = 4486)	<5 years (*n* = 11,220)	5–9 years (*n* = 9272)	10–19 years (*n* = 11,372)	20–29 years (*n* = 25,540)	30+ years (*n* = 17,944)
No. (%) with at least 1 MH-related ED visit	295 (10.52)	210 (9.06)	215 (7.56)	270 (4.23)	75 (1.67)	672 (5.99)	363 (3.92)	330 (2.99)	916 (3.59)	509 (2.84)
Mean no. visits (SD)^a^	2.75 (4.07)	2.81 (7.94)	1.85 (1.97)	1.92 (2.41)	1.63 (1.61)	1.95 (2.84)	1.94 (2.67)	1.66 (1.72)	1.75 (1.99)	1.69 (1.70)
Range	0-40	0-109	0-19	0-18	0-10	0-39	0-34	0-18	0-28	0-16
Total no. ED visits	811	590	398	519	122	1309	704	548	1599	861
Total no. person years of follow-up^b^	21,244	14,449	18,126	58,673	35,228	92,011	62,170	76,783	243,309	143,530
Crude rate of MH-related ED visits^c^	0.38	0.41	0.22	0.09	0.03	0.14	0.11	0.07	0.07	0.06
Crude rate ratio (veterans vs. non-veterans; ref. group = non-veterans)	2.71	3.73	3.14	1.29	0.50	1.00	1.00	1.00	1.00	1.00

*Note*. MH = mental health; ED = emergency department; SD = standard deviation; Ref. group = reference group.

^a^ Among individuals with at least one MH-related ED visit.

^b^ Rounded up to the nearest whole number.

^c^ Per 10 person-years of follow-up.

Overall, the reason for ED visits did not vary largely between veterans and non-veterans (see Supplemental Table S2). However, substance-related disorders accounted for a larger proportion of ED visits among female veterans compared to non-veterans (35.6% [*n* = 182] vs. 12.7% [*n* = 89]), but a smaller proportion of all MH-related ED visits among male veterans compared to non-veterans (23.8% [*n* = 459] vs. 32.5% [*n* = 1403]). Among veterans with 30+ years of service, mood and anxiety disorders accounted for a larger proportion of visits while substance-related disorders accounted for a smaller proportion of visits compared to non-veterans and veterans with fewer years of service (Supplemental Table S3).

**Figure 1.**
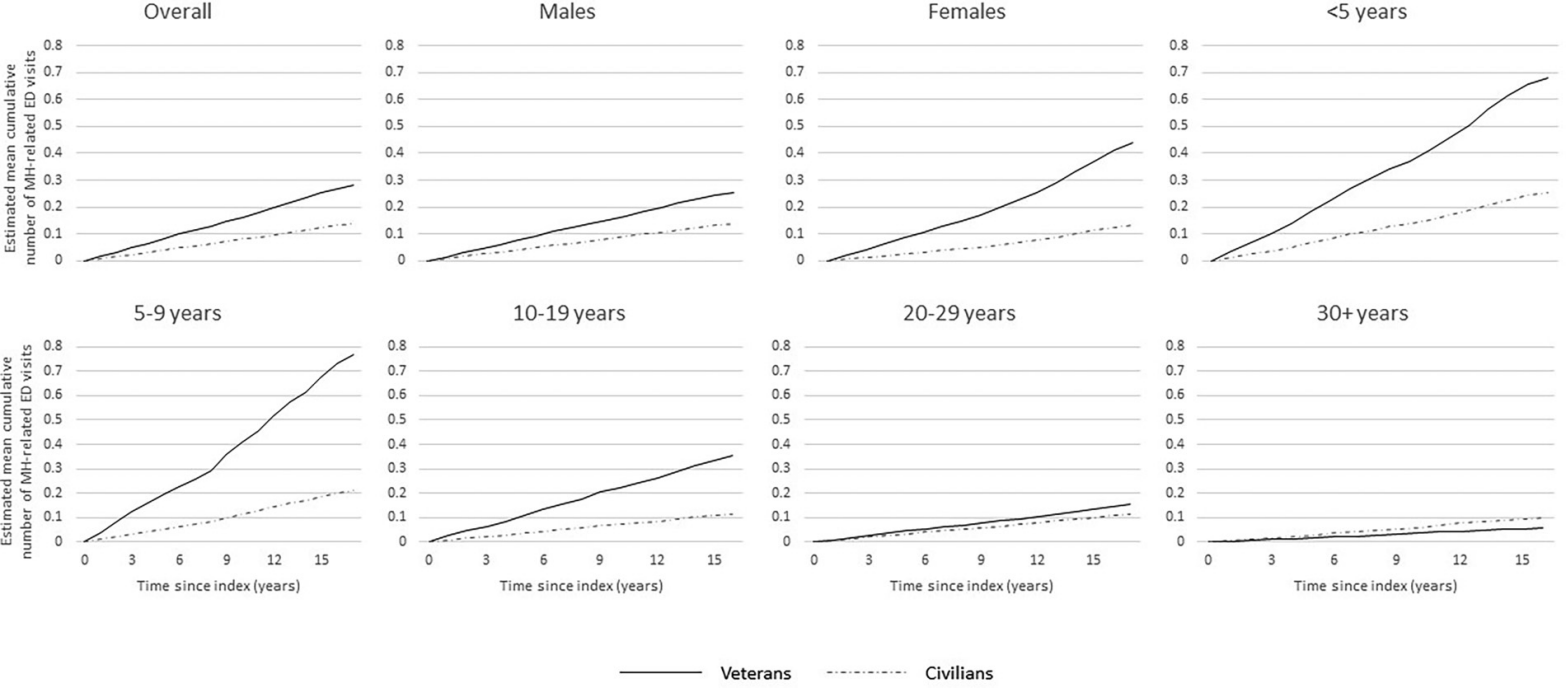
Estimated mean cumulative function for mental health-related emergency department (ED) visits over time among veterans and a matched non-veteran cohort, overall and stratified by sex and length of service.

The MCFs (Figure 1) demonstrate that, overall, veterans have a higher cumulative number of MH-related ED visits over time compared to non-veterans. Larger relative differences were observed for female veterans, and veterans with fewer years of service compared to non-veterans. Veterans with 30+ years of service had a lower cumulative number of MH-related ED visits over time compared to non-veterans.

**Figure 2.**
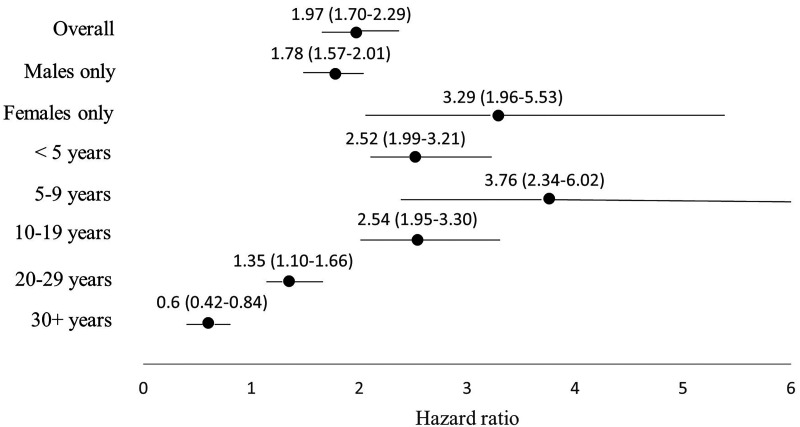
Adjusted hazard ratios and 95% confidence intervals for recurrent mental health-related emergency department (ED) visits among veterans residing in Ontario and a matched non-veteran cohort, overall and stratified by sex and length of service.

The interaction terms between veteran status and (1) sex and (2) length of service were statistically significant, suggesting effect measure modification by sex and length of service. Figure 2 shows the adjusted HRs (aHRs) and 95% confidence intervals (CIs) for the overall and sex- and length of service-stratified models. Overall, veterans had a higher rate of MH-related ED visits than non-veterans (aHR, 1.97; 95% CI, 1.70 to 2.29; *P* < 0.001). Relative differences were larger among females (aHR, 3.29; 95% CI, 1.96 to 5.53, *P* < 0.001) than males (aHR, 1.78; 95% CI, 1.57 to 2.01; *P* < 0.001). Veterans with fewer years of service had higher aHRs of MH-related ED visits than non-veterans (e.g., <5 years aHR, 2.52; 95% CI, 1.99 to 3.21, *P* < 0.001); only veterans with 30+ years of service had a lower aHR compared to non-veterans (aHR, 0.60; 95% CI, 0.42 to 0.84; *P* = 0.003).

## Discussion

This study demonstrated that, overall, veterans had a higher rate of MH-related ED visits than an age-, sex-, geography-, and income-matched cohort of non-veterans. This could be indicative of a greater need for acute MHC compared to non-veterans or reduced access to PCPs than non-veterans. Importantly, these rates varied by sex and length of service strata. Among females, veterans had significantly higher rates of MH-related ED visits than non-veterans; and the proportion of substance use-related ED visits was markedly higher among female veterans compared to non-veterans (35.6% vs. 12.7%, respectively), which is consistent with research from the US.^43,44^ Within veterans, a larger proportion of females had at least one MH-related ED visit compared to males, and a greater proportion of visits were attributable to substance use (35.6% vs. 23.8%, respectively). Previous research has documented that females are more likely to have a PCP and engage in healthcare-seeking behaviours than males,^45,46^ which may partially explain the sex-based differences observed in the estimated population mean cumulative number of MH-related ED visits among veterans. However, the same was not apparent among non-veterans, potentially signalling an interaction between veteran status and sex. Alcohol consumption may be a feature of male-dominated military culture (i.e., to boost morale, as a coping technique, etc.)^47^ that could be retained in the years following release or untreated substance misuse could lead to termination of military service, rendering this a potential sex-specific risk explaining the differences between female veterans and non-veterans observed here. These findings indicate that the provision of community-based interventions specifically targeting female veterans, such as harm reduction groups, may be of benefit to this subgroup of veterans.

This study also found that MH-related ED visits decreased among veterans with increasing years of service, and at 30+ years of service, it falls below the relatively stable rates observed among non-veterans across the length of service strata. This may be partially attributable to an interaction between time and the healthy worker effect.^48^ Military and policing careers are physically and mentally demanding, and individuals who are healthy enough to continue in these occupations may be healthier or less likely to have entered military service with an undiagnosed MH condition than individuals who serve for shorter periods of time.^49–53^ Indeed, research from Scotland suggests that veterans with <2.5 years of military service were more likely to receive a MH diagnosis following release than veterans who had served for longer periods,^17^ although this could be attributed to MH conditions going undiagnosed until it interfered with military service as well as conditions onsetting during the period of military service.^17,52,53^ Thus, the requirement of persistent good health in these occupations could result in higher rates of ED use among those who were more likely to be selected out of service for health-related reasons compared to those who remain in good health and are therefore able to serve longer. Alternatively, veterans with fewer years of service may be younger and less likely to access primary care upon release than longer-serving veterans, as seen in other research examining medical services use among veterans in Ontario,^54^ leaving the ED as the initial point of contact on the pathway to continuous MHC. While all veterans exiting the CAF are provided with information about pathways to MHC, recommendations are not currently provided based on length of service. As such, increased psychoeducation and assistance connecting with PCPs upon release from the CAF or RCMP may help reduce the number of MH-related ED visits among early service-leavers.

The current study builds on previous research suggesting that the rate of MH-related ED visits remains relatively steady over a 20-year period following release from the CAF or the RCMP^55^ by facilitating comparisons to a matched cohort of non-veterans overall and by sex and length of service. Our study uses population-level data and likely includes most veterans residing in Ontario,^56^ but has some limitations. First, because the MOH uses a single veteran identifier, we are unable to explore MH-related ED use for CAF and RCMP veterans separately. The inclusion of RCMP veterans, who comprise an estimated 15% of our veteran cohort,^56^ may restrict comparability with research from other provinces or nations. It is also possible that unmeasured variables, such as history of trauma, family or childhood history of MH diagnoses, and primary care access, could have resulted in an overestimation of the relationships between veteran status and MH-related ED use. We acknowledge that the index date used in this study carries more importance for veterans than non-veterans; and that the matching and restriction approaches used in the creation of the non-veteran cohort resulted in a comparison group of non-veterans who may be more similar to the veteran cohort than the Ontario general population. Further, a slightly larger proportion of veterans were censored by moving out of province than non-veterans. However, individuals continue to be at risk for the outcome even after censoring, meaning that we may have underestimated true rates of MH-related ED visits. Differences in rurality between veterans, who were more likely to live in smaller urban centres or rural communities, and non-veterans, who were more likely to live in a densely population urban area, may also influence the use of EDs for MH concerns. Veterans may have access to additional non-urgent MH services compared to non-veterans, such as the VAC-funded network of Operational Stress Injury Clinics, which may reduce the need to access EDs to initiate MHC. Finally, we are unable to ascertain whether our findings are the result of increased MH needs, increased healthcare-seeking behaviours, reduced access to PCPs, or the joint effect of these mechanisms.

## Conclusions

This research builds on our existing knowledge of MH services use among veterans by comparing rates of MH-related ED use to non-veterans in Ontario overall, and by sex and length of service. This work highlights heterogeneity in rates of MH-related ED visits, with increased rates observed among female veterans and veterans with fewer years of service. These findings provide new knowledge related to the health of Canadian veterans—1 of VAC's 7 domains of well-being^57^—and emphasize the importance of looking beyond overall rates for healthcare planning and delivery purposes at the provincial level and for veteran health administrations, who offer additional avenues for healthcare services to veterans. Additionally, the methods used in this study could be applied to comparative MH services use research in other provinces and countries. Future work incorporating triage acuity and primary diagnostic codes, or linkages to VAC databases, could help further identify veterans who might benefit from targeted, community-based MHC.

## Supplemental Material

sj-docx-1-cpa-10.1177_07067437231223328 - Supplemental material for A Retrospective Cohort Analysis of Mental Health-Related Emergency Department Visits Among Veterans and Non-Veterans Residing in Ontario, Canada: Une analyse de cohorte rétrospective des visites au service d’urgence liées à la santé mentale parmi les vétérans et non-vétérans résidant en Ontario, Canada Supplemental material, sj-docx-1-cpa-10.1177_07067437231223328 for A Retrospective Cohort Analysis of Mental Health-Related Emergency Department Visits Among Veterans and Non-Veterans Residing in Ontario, Canada: Une analyse de cohorte rétrospective des visites au service d’urgence liées à la santé mentale parmi les vétérans et non-vétérans résidant en Ontario, Canada by Kate St. Cyr, Peter Smith, Paul Kurdyak, Heidi Cramm, Alice B. Aiken and Alyson Mahar in The Canadian Journal of Psychiatry
