# Supplementary material for: A Retrospective Cohort Analysis of Mental Health-Related Emergency Department Visits Among Veterans and Non-Veterans Residing in Ontario, Canada: Une analyse de cohorte rétrospective des visites au service d’urgence liées à la santé mentale parmi les vétérans et non-vétérans résidant en Ontario, Canada
Source: Can J Psychiatry. 2024 Jan 5;69(5):347–57. doi: 10.1177/07067437231223328 (PMC11032094; doi:10.1177/07067437231223328)
Supplement: sj-docx-1-cpa-10.1177_07067437231223328 - Supplemental material for A Retrospective Cohort Analysis of Mental Health-Related Emergency Department Visits Among Veterans and Non-Veterans Residing in Ontario, Canada: Une analyse de cohorte rétrospective des visites au service d’urgence liées à la santé [file sj-docx-1-cpa-10.1177_07067437231223328.docx]

**Supplemental Table 1: ICD-10 categories used in ICES dx groupings**

| **ICES dx grouping** | **International Classification of Diseases and Related Health Problems, 10^th^ revision (ICD-10), Canadian Enhancement Codes** |
| --- | --- |
| Anxiety disorders | F40: Phobic anxiety disorders  F41: Other anxiety disorders  F42: Obsessive-compulsive disorder  F43: Reaction to severe stress and adjustment disorders  F48.8: Other specified neurotic disorders  F48.9: Neurotic disorder, unspecified |
| Deliberate self-harm | X60: Intentional self-poisoning by and exposure to non-opioid  analgesics, antipyretics and antirheumatics  X61: Intentional self-poisoning by and exposure to antiepileptic, sedative-hypnotic, antiparkinsonism and psychotropic drugs, NOS  X62: Intentional self-poisoning by and exposure to narcotics and psychodysleptics [hallucinogens], NOS  X63: Intentional self-poisoning by and exposure to other drugs acting on the autonomic nervous system  X64: Intentional self-poisoning by and exposure to other and unspecified drugs, medicaments and biological substances  X65: Intentional self-poisoning by and exposure to alcohol  X66: Intentional self-poisoning by and exposure to organic solvents and halogenated hydrocarbons and their vapours  X67: Intentional self-poisoning by and exposure to other gases and vapours  X68: Intentional self-poisoning by and exposure to pesticides  X69: Intentional self-poisoning by and exposure to other and unspecified chemicals and noxious substances  X70: Intentional self-harm by hanging, strangulation and suffocation  X71: Intentional self-harm by drowning and submersion  X72: Intentional self-harm by handgun discharge  X73: Intentional self-harm by rifle, shotgun and larger firearm discharge  X74: Intentional self-harm by other and unspecified firearm discharge  X75: Intentional self-harm by explosive material  X76: Intentional self-harm by smoke, fire and flames  X77: Intentional self-harm by steam, hot vapours and hot objects  X78: Intentional self-harm by sharp object  X79: Intentional self-harm by blunt object  X80: Intentional self-harm by jumping from a high place  X81: Intentional self-harm by jumping or lying before a moving object  X82: Intentional self-harm by crashing of motor vehicle  X83: Intentional self-harm by other specified means  X84: Intentional self-harm by unspecified means  Y10: Poisoning by and exposure to non-opioid analgesics, antipyretics  and antirheumatics, undetermined intent  Y11: Poisoning by and exposure to antiepileptic, sedative-hypnotic, antiparkinsonism and psychotropic drugs, not elsewhere classified, undetermined intent  Y12: Poisoning by and exposure to narcotics and psychodysleptics [hallucinogens], not elsewhere classified, undetermined intent  Y13: Poisoning by and exposure to other drugs acting on the autonomic nervous system, undetermined intent  Y14: Poisoning by and exposure to other and unspecified drugs,  medicaments and biological substances, undetermined intent  Y15: Poisoning by and exposure to alcohol, undetermined intent  Y16: Poisoning by and exposure to organic solvents and halogenated hydrocarbons and their vapours, undetermined intent  Y17: Poisoning by and exposure to other gases and vapours,  undetermined intent  Y18: Poisoning by and exposure to pesticides, undetermined intent  Y19: Poisoning by and exposure to other and unspecified chemicals  and noxious substances, undetermined intent  Y28: Contact with sharp object, undetermined intent |
| Mood disorders | F30: Manic episode  F31: Bipolar affective disorder  F32: Depressive episode  F33: Recurrent depressive disorder  F34: Persistent mood [affective] disorders  F38: Other mood [affective] disorders  F39: Unspecified mood [affective] disorder  F53.0: Mild mental and behavioural disorders associated with the puerperium, not elsewhere classified |
| Schizophrenia and other psychotic disorders | F20: Schizophrenia (excluding F20.4: Post-schizophrenic depression)  F22: Persistent delusional disorders  F23: Acute and transient psychotic disorders  F24: Induced delusional disorder  F25: Schizoaffective disorders  F28: Other nonorganic psychotic disorders  F29: Unspecified nonorganic psychosis  F53.1: Severe mental and behavioural disorders associated with the puerperium, not elsewhere classified |
| Substance-related disorders | F10: Mental and behavioural disorders due to use of alcohol  F11: Mental and behavioural disorders due to use of opioids  F12: Mental and behavioural disorders due to use of cannabinoids  F13: Mental and behavioural disorders due to use of sedatives or hypnotics  F14: Mental and behavioural disorders due to use of cocaine  F15: Mental and behavioural disorders due to use of other stimulants, including caffeine  F16: Mental and behavioural disorders due to use of hallucinogens  F17: Mental and behavioural disorders due to use of tobacco  F18: Mental and behavioural disorders due to use of volatile solvents  F19: Mental and behavioural disorders due to multiple drug use and use of other psychoactive substances  F55: Abuse of non-dependence-producing substances |

**Supplemental Table 2. Reason for all MH-related ED visits during period of follow-up (April 1, 2002 to March 31, 2020), by Veteran status overall and by sex**

| **ICES dx grouping** | **No. (% of all MH-related ED visits during follow-up period)** | | | | | |
| --- | --- | --- | --- | --- | --- | --- |
|  | **Veterans** | | | **Non-Veterans** | | |
|  | **Overall (No. visits = 2440)** | **Males (No. = 1929)** | **Females (No. = 511)** | **Overall (No. visits = 5021)** | **Males (No. = 4322)** | **Females (No. = 699)** |
| Anxiety disorders | 904 (37.05) | 742 (38.47) | 162 (31.70) | 1,895 (37.73) | 1,569 (36.29) | 326 (46.64) |
| Deliberate self-harm | 159 (6.52) | 131 (6.79) | 28 (5.48) | 315 (6.27) | 269 (6.22) | 46 (6.58) |
| Mood disorders | 486 (19.92) | 390 (20.22) | 96 (18.79) | 877 (17.46) | 710 (16.42) | 167 (23.89) |
| Schizophrenia and other psychotic disorders | 135 (5.53) | 125 (6.48) | 10 (1.96) | 256 (5.10) | 227 (5.25) | 29 (4.15) |
| Substance-related disorders | 641 (26.27) | 459 (23.80) | 182 (35.62) | 1,493 (29.73) | 1,403 (32.48) | 89 (12.73) |
| Other^a^ | 115 (4.71) | 82 (4.25) | 33 (6.46) | 186 (3.70) | 144 (3.33) | 42 (6.01) |

^a^Other refers diagnostic codes not captured by the other categories including, but not limited to, personality disorders, somatoform disorders, and mild cognitive disorders

**Supplemental Table 3. Reason for all MH-related ED visits during period of follow-up (April 1, 2002 to March 31, 2020), by Veteran status overall and length of service**

| **ICES dx grouping** | **No. (% of all MH-related ED visits during follow-up period)** | | | | | | | | | |
| --- | --- | --- | --- | --- | --- | --- | --- | --- | --- | --- |
|  | **Veterans** | | | | | **Non-Veterans** | | | | |
|  | **< 5 years (No. visits = 811)** | **5-9 years (No. visits = 590)** | **10-19 years (No. visits = 398)** | **20-29 years (No. visits = 519)** | **30+ years (No. visits = 122)** | **< 5 years (No. visits = 1309)** | **5-9 years (No. visits = 704)** | **10-19 years (No. visits = 548)** | **20-29 years (No. visits = 1599)** | **30+ years (No. visits = 861)** |
| Anxiety disorders | 251 (30.95) | 210 (35.59) | 168 (42.21) | 216 (41.62) | 59 (48.36) | 426 (32.54) | 289 (41.05) | 257 (46.90) | 641 (40.09) | 282 (32.75) |
| Deliberate self-harm | 59 (7.27) | 35 (5.93) | 24 (6.03) | 32 (6.17) | -^b^ | 79 (6.04) | 40 (5.68) | 35 (6.39) | 110 (6.88) | 51 (5.92) |
| Mood disorders | 142 (17.51) | 95 (16.10) | 83 (20.85) | 135 (26.01) | 31 (25.41) | 230 (17.57) | 99 (14.06) | 94 (17.15) | 286 (17.89) | 168 (19.51) |
| Schizophrenia and other psychotic disorders | 77 (9.49) | 19 (3.22) | 19 (4.77) | 18 (3.47) | -^b^ | 104 (7.94) | 37 (5.26) | 33 (6.02) | 56 (3.50) | 26 (3.02) |
| Substance-related disorders | 225 (27.74) | 219 (37.12) | 74 (18.59) | 107 (20.62) | 16 (13.11) | 426 (32.54) | 215 (30.54) | 111 (20.26) | 437 (27.33) | 303 (35.19) |
| Other^a^ | 57 (7.03) | 12 (2.31) | 30 (7.54) | 11 (2.12) | -^b^ | 44 (3.36) | 24 (3.41) | 18 (3.28) | 69 (4.32) | 31 (3.60) |

^a^Other refers diagnostic codes not captured by the other categories including, but not limited to, personality disorders, somatoform disorders, and mild cognitive disorders; ^b^cells are suppressed due to small sizes.
